# Silibinin inhibits accumulation of myeloid-derived suppressor cells and tumor growth of murine breast cancer

**DOI:** 10.1002/cam4.186

**Published:** 2014-01-20

**Authors:** Parvin Forghani, Mohammad R Khorramizadeh, Edmund K Waller

**Affiliations:** 1 Department of Pathobiology, School of Public Health, Tehran University of Medical SciencesTehran, Iran; 2 Department of Hematology and Medical Oncology, Winship Cancer Institute, Emory UniversityAtlanta, Georgia; 3 School of Public Health, Department of Medical Biotechnology, School of Advanced Medical Technologies Tehran University of Medical SciencesTehran, Iran

**Keywords:** 4T1, breast cancer, mice, myeloid-derived suppressor cells, SCID, silibinin

## Abstract

Myeloid-derived suppressor cells (MDSC)s increase in blood and accumulate in the tumor microenvironment of tumor-bearing animals, contributing to immune suppression in cancer. Silibinin, a natural flavonoid from the seeds of milk thistle, has been developed as an anti-inflammatory agent and supportive care agent to reduce the toxicity of cancer chemotherapy. The goals of this study were to evaluate the effect of silibinin on MDSCs in tumor-bearing mice and antitumor activity of silibinin in a mouse model of breast cancer. 4T1 luciferase-transfected mammary carcinoma cells were injected into in the mammary fat pad female BALB/c mice, and female CB17-Prkdc Scid/J mice. Silibinin treatment started on day 4 or day 14 after tumor inoculation continued every other day. Tumor growth was monitored by bioluminescent imaging (BLI) measuring total photon flux. Flow cytometry measured total leukocytes, CD11b^+^ Gr-1^+^ MDSC, and T cells in the blood and tumors of tumor-bearing mice. The effects of silibinin on 4T1 cell viability in vitro were measured by BLI. Treatment with silibinin increased overall survival in mice harboring tumors derived from the 4T1-luciferase breast cancer cell line, and reduced tumor volumes and numbers of CD11b^+^Gr-1^+^ MDSCs in the blood and tumor, and increased the content of T cells in the tumor microenvironment. Silibinin failed to inhibit tumor growth in immunocompromised severe combined immunodeficiency mice, supporting the hypothesis that anticancer effect of silibinin is immune-mediated. The antitumor activity of silibinin requires an intact host immune system and is associated with decreased accumulation of blood and tumor-associated MDSCs.

## Introduction

Studies in animal models have been shown that a variety of suppressor cells in the tumor-bearing host constitute an immunosuppressive network that inhibits antitumor immunity 1,2_._ Myeloid-derived suppressor cells (MDSC)s are potent suppressor cells, defined phenotypically in mice as myeloid progenitors that co-express CD11b^+^ and Gr-1^+^ 3–6. Preclinical studies have shown a correlation between tumor progression and accumulation of MDSCs in a variety of cancer types 3,4. Given recent discoveries surrounding the function and involvement of MDSCs in cancer promotion and immune evasion, MDSCs are now recognized as key mediators of immunosuppression in cancer 1,7–9.

An association between the development of cancer and inflammation was proposed by the German pathologist Rudolf Virchow over 140 years ago 10. Several findings support the role for inflammation as a predisposition for the development of cancer 11,12. Inflammation promotes malignant cell growth by inducing immune suppression 13. It has been shown that decreased accumulation of MDSCs is one of the mechanisms by which reducing inflammation may facilitate immune surveillance and antitumor immunity 14. With this background, we sought to identify whether silibinin, a natural compound with anti-inflammatory activity, could modulate the content of MDSCs in tumor-bearing mice and enhance antitumor immune responses.

Silibinin, is a bioactive flavonolignan extracted from the blessed milk thistle 15. Many studies have shown that silibinin inhibits experimentally induced malignancies of the prostate, skin, and colon 15–18 as well as inhibition of proliferation of cancer cell lines in vitro 19–23. Silibinin has been evaluated in clinical trials as a chemo-protective agent in cancer patients 24,25. However, exact mechanisms of the antitumor effects of silibinin have not been clearly elucidated 26. We hypothesized that the reported antitumor activity of silibinin may be secondary to the suppression of tumor-induced inflammation resulting in decreased accumulation of immunosuppressive MDSCs. Here we tested the biological effect of silibinin on tumor cells and MDSCs in an in vivo model of murine breast cancer. We evaluated the effect of silibinin treatment on the accumulation of MDSCs in the blood and tumor tissue of immunocompetent BALB/c versus immunecompromised severe combined immunodeficiency (SCID) mice bearing 4T1 tumors. Bioluminescent imaging (BLI) and tumor measurements in live animals harboring luciferase-transfected tumors were used to monitor tumor burden at multiple time points during the course of tumor growth. Taken together, the results of this study indicate a new mechanism for the antitumor effect of silibinin as an immunotherapeutic anticancer agent that inhibits tumor-associated accumulation of MDSCs.

## Methods

### Mice and tumor model

Female BALB/c and SCID CB17-Prkdcscid/J mice (age 6–8 weeks) were purchased from Jackson Laboratories, and maintained according to the IUCAC Guidelines of Emory. 4T1, a BALB/c mouse breast cancer cell line was obtained from ATCC (Manassas, VA). The protocol was approved by the committee on the Ethics of animal Experiments of the University of Emory (Permit number: A3180-01). Stably transfected luciferase-expressing 4T1 was a gift of Dr. Lili Yang. 4T1 cell lines were cultured in RPMI 1640 (Life Technologies, Grand Island, NY) and Dulbecco's modified Eagle's medium (DMEM/hi glucose) supplemented with 10% fetal bovine serum enriched with 0.4 mmol/L of sodium pyruvate, and antibiotics (penicillin, streptomycin), respectively. Female BALB/c or SCID mice were injected subcutaneously in the left 1rd mammary fat pad with 1 × 10^6^ cells viable 4T1 tumor cells in 100 *μ*L phosphate-buffered saline (PBS). Mice were monitored every other day to evaluate tumor growth. Treatment of tumor-bearing mice began when tumor growth was confirmed with BLI.

### Tumor measurements

4T1 tumor-bearing BAlB/c mice were anesthetized with diluted ketamine/xylazine based on Emory ICACUC guidelines i.p. Synthetic firefly d-luciferin potassium salt stock solutions (15 mg/mL in PBS) were prepared immediately before imaging, and then injected s.c. at a dose of 150 mg/kg 27. Imaging was performed at multiple time points in anesthetized mice using an IVIS 100 charge-coupled device imaging system (Xenogen, Alameda, CA). Imaging data were analyzed with Living Image Version 3.2 software (Alameda, CA). Bioluminescence intensity regions of interest (ROI)s were displayed in photons mode (unit is photons/s). Images were compared using the same BLI intensity scale and setting (field of view: 25, binning: medium, f stop: 1, exposure time: sequential pattern). In order to determine tumor volume, the greatest longitudinal diameter (length) and the greatest transverse diameter (width) were determined using calipers. Tumor volume based on caliper measurements was calculated by the modified ellipsoidal formula 28: Tumor volume* *= 1/2 (length × width^2^).

### In vivo and in vitro treatments

Mice with palpable tumors or tumor detectable by BLI were randomly divided into two groups of four to five mice. Treatment groups received silibinin (Sigma, St. Louis MO) at 150 mg/kg of body weight by gavage in a vehicle consisting of 0.05% (w/v) Carboxymethyl cellulose (CMC) and Triton X-100, for 4–5 weeks starting 4 or 14 days after tumor inoculation. Control mice were gavaged three times/week with vehicle alone. For silibinin treatment in vitro, appropriate amounts of stock solution (500 *μ*mol/L in dimethyl sulfoxide) of silibinin were added into in 96-well plates in serial dilutions to achieve the indicated final concentrations and then incubated with cells for 24 h. The detection sensitivity of BLI on cells in vitro was determined by measuring the bioluminescent signal emitted from known numbers of cells in culture using an IVIS imaging system. ROI measured with the specified exposure time following the addition of luciferin (Gold Biotechnology, St Louis, MO) at a final concentration of 150 mg/mL in the cell culture media.

### Isolation of MDSCs from tumors and blood

Implanted tumors were mechanically dissociated into suspensions of individual spleen or tumor cells expressing tissue fragments through a 70-*μ*m cellular sieve with a plunger. Cell suspensions were centrifuged, counted, and washed once with PBS. Blood samples were drawn from the tail vein according to IACUC Guidelines. Peripheral blood was counted using a Coulter AcT diff Analyzer (Beckman, Miami, FL).

### Flow cytometry analysis

Blood leukocytes and single-cell suspensions of tumors were stained with allophycocyanin (APC)-cyanine7 (Cy7)-conjugated anti-CD11b, fluorescein isothiocyanate (FITC)-conjugated Gr-1, phycoerythrine-conjugated lineage markers, and Fluorescence-Activated Cell Sorting (FACS) for 20 min in 4°C as we have previously described 29. All antibodies were purchased from BD PharMingen or eBioscience (San Jose, CA). Quantitative determination of cell counts in tumor suspensions and blood was determined using an AccuCheck Counting Beads (Invitrogen, Carlsbad, CA) added to samples with a known volume. The absolute count (cells/*μ*L) was calculated using the equation (cells/*μ*L = number of cells counted/total number of beads counted × concentration). For intracellular cytokine staining, cell suspensions were washed with cold PBS, permeabilized according to the manufacturer's protocol (Fix/Perm eBioscience), and stained with an anti-CD206, TNF*α*, IL-1*β*, and IL-10 antibodies (eBiosciences, San Diego, CA) for 20 min at 4°C. CC chemokine receptor 2 (CCR)2 and CCR7 staining was performed at room temperature for 20 min. Following a final wash with cold PBS, cells were analyzed by flow cytometry using FACS Diva software (BD Biosciences, San Jose, CA) and FlowJo software (TreeStar, Ashland, OR).

### Statistical analysis

Student's *t*-test or Mann–Whitney test was used for parametric and nonparametric means comparison. Log-rank (mantel-cox) test used to compare survival curves. All tests were performed with Graph Pad Prism software (San Diego, CA).

## Results and Discussion

### Silibinin treatment delayed growth of 4T1 and prolonged survival in 4T1 tumor-bearing mice

**Figure 1:**
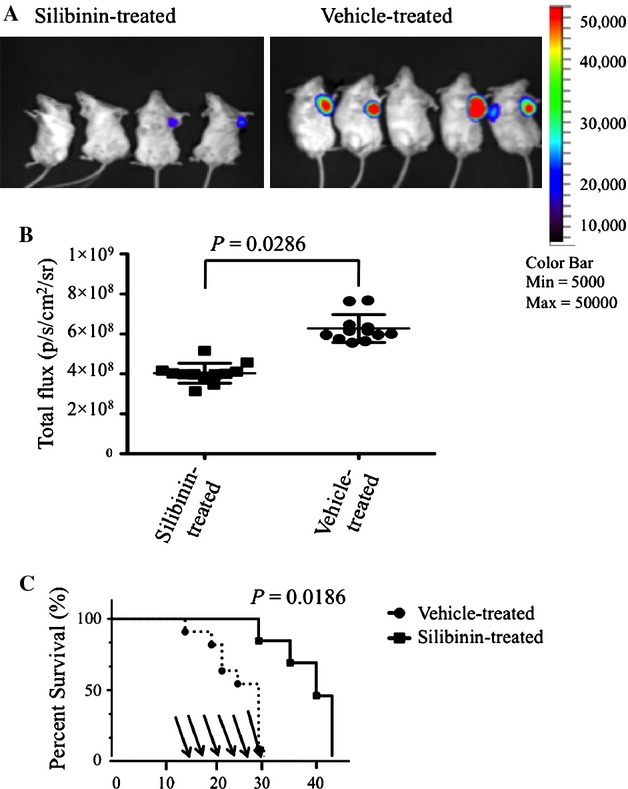
Silibinin treatment decreased tumor volume and increased survival in mice with 4T1 breast cancer tumors. BALB/c mice were inoculated with 1 × 10^6^ 4T1-luciferase tumor cells and then treated with silibinin by gavage three times per week starting on day 14 (150 mg/kg). (A) Pseudo color luminescent images represent increasing emitted light and tumor volumes (blue, green, yellow, and red from least to most intense, respectively). Representative silibinin-treated versus vehicle-treated mice. In vivo visualization of 4T1 breast tumor volume at day 28 after tumor inoculation. (B) Total flux detected in individual mice after silibinin treatment on day 28 after tumor inoculation comparing silibinin-treated and control groups. Total flux collected in an ROI following a 30-sec exposure time. (C) Survival of silibinin-treated BALB/c mice versus vehicle-treated BALB/c mice. The times of gavage are shown as arrows. Data are compiled from three separate experiments (*n* = 4–5 per group).

To determine whether silibinin inhibits 4T1 breast tumor growth, mice were inoculated subcutaneously with 4T1-luc cells in the left flank. Although tumors were not palpable until day 7 post implantation, bioluminescent signals were detected from day 0 (data not shown). Two weeks after tumor inoculation, groups of mice were treated with vehicle (control) or silibinin (150 mg/kg) every other day by gavage. Comparing silibinin-treated mice versus vehicle-treated mice, a significant decrease in total flux was seen following six treatments over 14 days. (Fig. 1A and B; *P *<* *0.05). Animals were then followed up for survival following the 14 days of treatment with silibinin or vehicle. Silibinin treatment was associated with a significant prolongation of survival compared with vehicle-treated controls, with a median survival of 34 days versus 25 days, respectively. (Fig. 1C; *P *<* *0.05). Physical measurement of tumor volume with calipers confirmed difference seen by BLI (data not shown).

### Silibinin treatment reduces the quantity of blood-derived MDSCs in tumor-bearing mice

**Figure 2:**
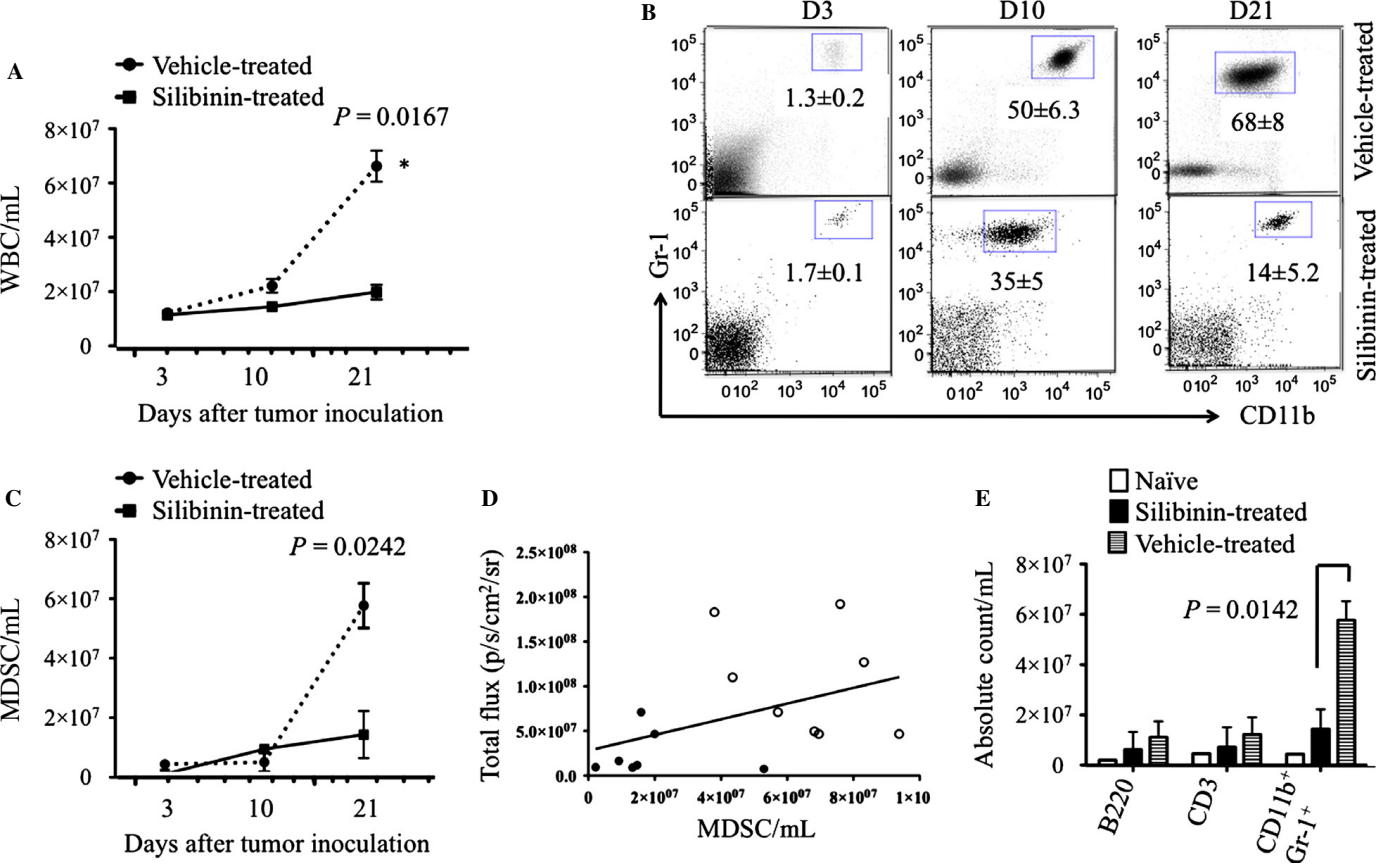
Silibinin treatment reduced the numbers of MDSCs in 4T1 tumor-bearing mice. 4T1 tumor cells were implanted into the first left mammary fat pad of female BALB/c mice (1 × 10^6^ cells per mouse, *n* = 4–5/group). Silibinin treatment was started on day 4 after tumor inoculation. (A) Blood samples were obtained on 3, 7, and 21 days of tumor inoculation and total leukocytes were counted. (B) Flow cytometry analysis of 7-AAD negative-gated CD11b^+^ Gr-1^+^ MDSCs in blood of 4T1-tumor-bearing mice, showing mean frequencies (±SD) of blood MDSCs in vehicle- and silibinin-treated groups from three separate experiments. (C) Absolute numbers of MDSCs in the blood after tumor inoculation in two groups. (D) Correlation between total fluxes of photon from tumor cells and absolute number of MDSCs at day 21 in vehicle-treated group (blank circle) versus silibinin-treated group (filled circle) (*R*^2^ = 0.08953). (E) The percentages of MDSCs, CD3^+^ T cells and CD220^+^ B cells in blood, comparing vehicle-treated control group and silibinin-treated mice on day 21 after tumor inoculation. *Statistically significant differences from control (*P *<* *0.05) compiled from three independent experiments.

To analyze the impact of silibinin treatment on blood leukocytes and MDSCs in tumor-bearing animals, mice were periodically bled and the numbers of blood leukocytes monitored after tumor inoculation. As previously reported using the 4T1 tumor model 30, tumor growth was associated with marked leukocytosis in the blood. The silibinin-treated group had significantly fewer blood leukocytes on day 21 after tumor inoculation compared to vehicle-treated controls (Fig. 2A). We next analyzed BM, blood, and tumor-infiltrating cells of tumor-bearing mice for the presence of CD11b^+^Gr-1^+^ lineage negative MDSCs. The frequency of MDSCs increased in blood of 4T1-tumor-bearing mice during tumor growth (Fig. 2B top panels). Similar increases were seen in the frequencies of MDSCs in BM, spleen, and the tumor in mice inoculated with 4T1 (data not shown). We then tested the effect of in vivo administration of silibinin on MDSCs accumulation in the blood. Administration of silibinin decreased the percentage and absolute numbers of MDSCs in the blood compared to vehicle-treated controls at serial time points (Fig. 2B, bottom panels and 2C). As shown in Figure 2C, the greatest decrease in the MDSCs content of the blood following silibinin treatment was seen 21 days after tumor inoculation. We found a linear association between the content of MDSCs in the blood of individual mice compared with tumor volume measured by BLI, supporting the idea, that generation and accumulation MDSCs are direct results of factors produced by the tumor/tumor microenvironment (Fig. 2D). To determine if the effect of silibinin on the composition of leukocytes in the blood was specific to the content of MDSCs, blood samples from silibinin-treated mice versus tumor-free mice and vehicle-treated BALB/c mice were analyzed for B cells, T cells, and MDSCs on day 21 after tumor inoculation. Tumor-bearing mice had significantly more MDSCs compared to vehicle-treated mice but no significant difference in the numbers of T cells or B cells was seen (Fig. 2E).

**Figure 3:**
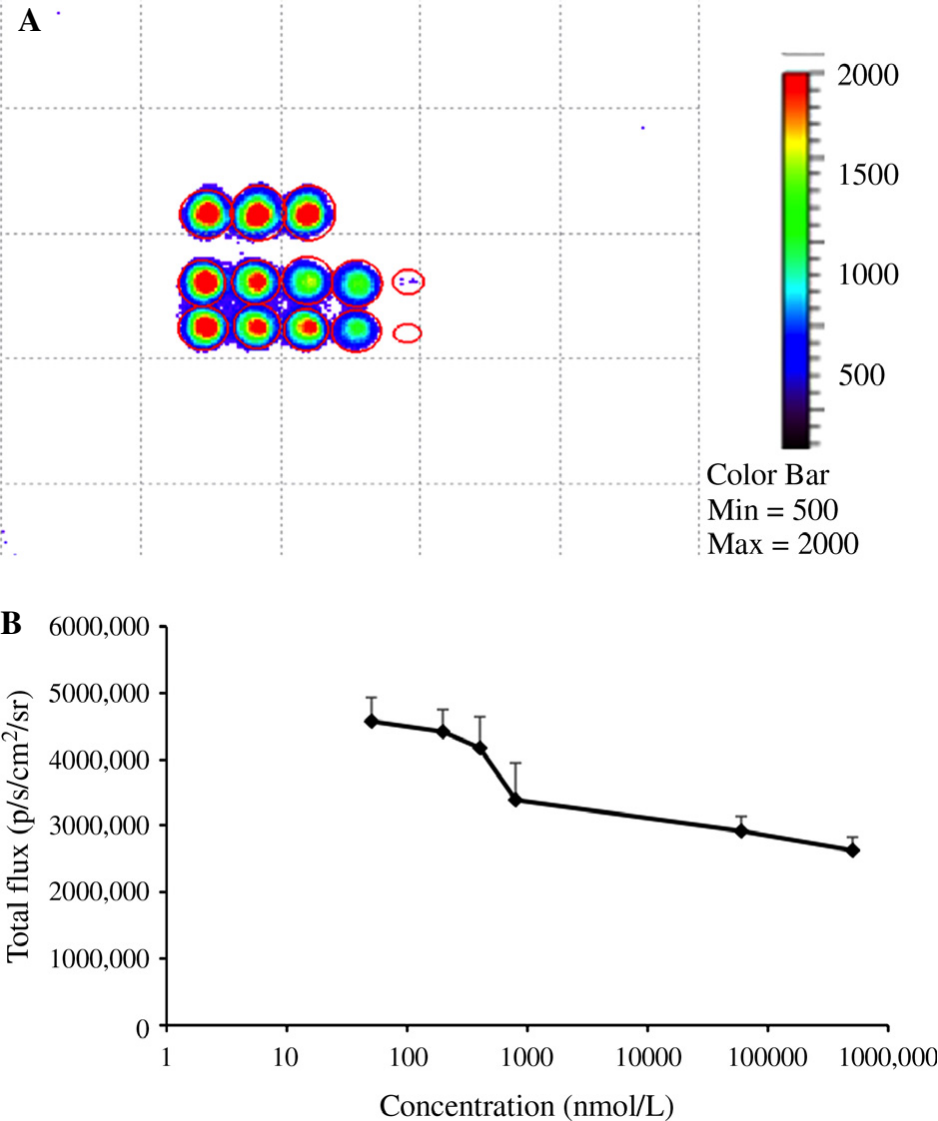
Silibinin has limited in vitro antiproliferative activity in 4T1 cells 4T1-luc tumor cells were seeded in triplicates in 96-well plates at a concentration of 10^5^–10^6^/well with media containing silibinin. The top row represents untreated cells in triplicate, and the two bottom rows represent treated cells in increasing concentrations of silibinin from left to right in duplicate. After 24 h luciferin was added to the medium and ROI measured in each well. (A) Graph represents total flux collected in well from three independent experiments. (B) Logarithmic graph represents of total flux of tumors versus different concentrations of silibinin. The horizontal axis shows concentration of silibinin.

Given evidence that MDSCs development begins in the bone marrow microenvironment 29, we evaluated the number of MDSCs in BM from nontumor-bearing mice after prolonged administration (2 months) of silibinin treatment. Treatment began every other day for the first month and then weekly for a second month. Contrary to the significant reduction in MDSCs numbers in the blood of silibinin-treated tumor-bearing mice, we did not find any significant difference in the numbers of MDSCs in BM or blood of nontumor-bearing mice following silibinin treatment (data not shown). Silibinin has been shown to have direct cytotoxic effects on tumor cells in vitro 21,31,32. To explore the mechanism by which silibinin decreased tumor growth in mice, we tested whether silibinin has a direct effect on 4T1 cell viability in vitro. 4T1 cells were treated at 60–70% confluency with different concentrations of silibinin for 24 h. Tumor viability was measured by BLI, as luciferase activity represents the number and viability of metabolically active 4T1–luciferase cells, and luciferase activity was not observed in apoptotic 4T1 cells or lysate prepared from 4T1 cells. On the basis of total flux, the metabolic activity of viable tumor cell line showed only a modest decrease following treatment with graduated levels of silibinin (Fig. 3 A and B).

### Silibinin did not inhibit tumor growth in 4T1-bearing immunocompromised SCID

**Figure 4:**
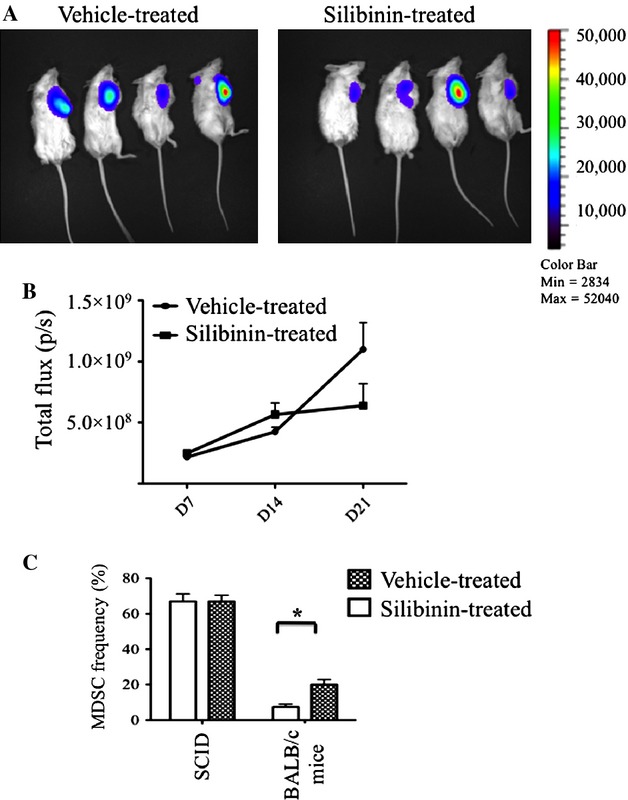
Silibinin treatment did not inhibit tumor growth in immunocompromised SCID mice. Immunocompromised SCID mice were inoculated with 1 × 10^6^ 4T1-luc cells s.c. Thrice weekly silibinin treatment was started on day 4 and tumor growth monitored by BLI. (A) BLI of mice representative from three experiments of tumor growth from silibinin-treated (right) and control group (left) showing 14 days after tumor inoculation. (B) Total flux from implanted tumor in silibinin-treated and control mice, exposure time 60 sec, (aggregate data from three replicate experiments, *n* = 4–5 per group in different time points. (C) Frequencies of MDSCs in the tumor microenvironment of SCID versus immunocompetent BALB/c mice, comparing silibinin-treated versus control vehicle-treated groups on day 25 after tumor inoculation. *P < 0.05.

**Figure 5:**
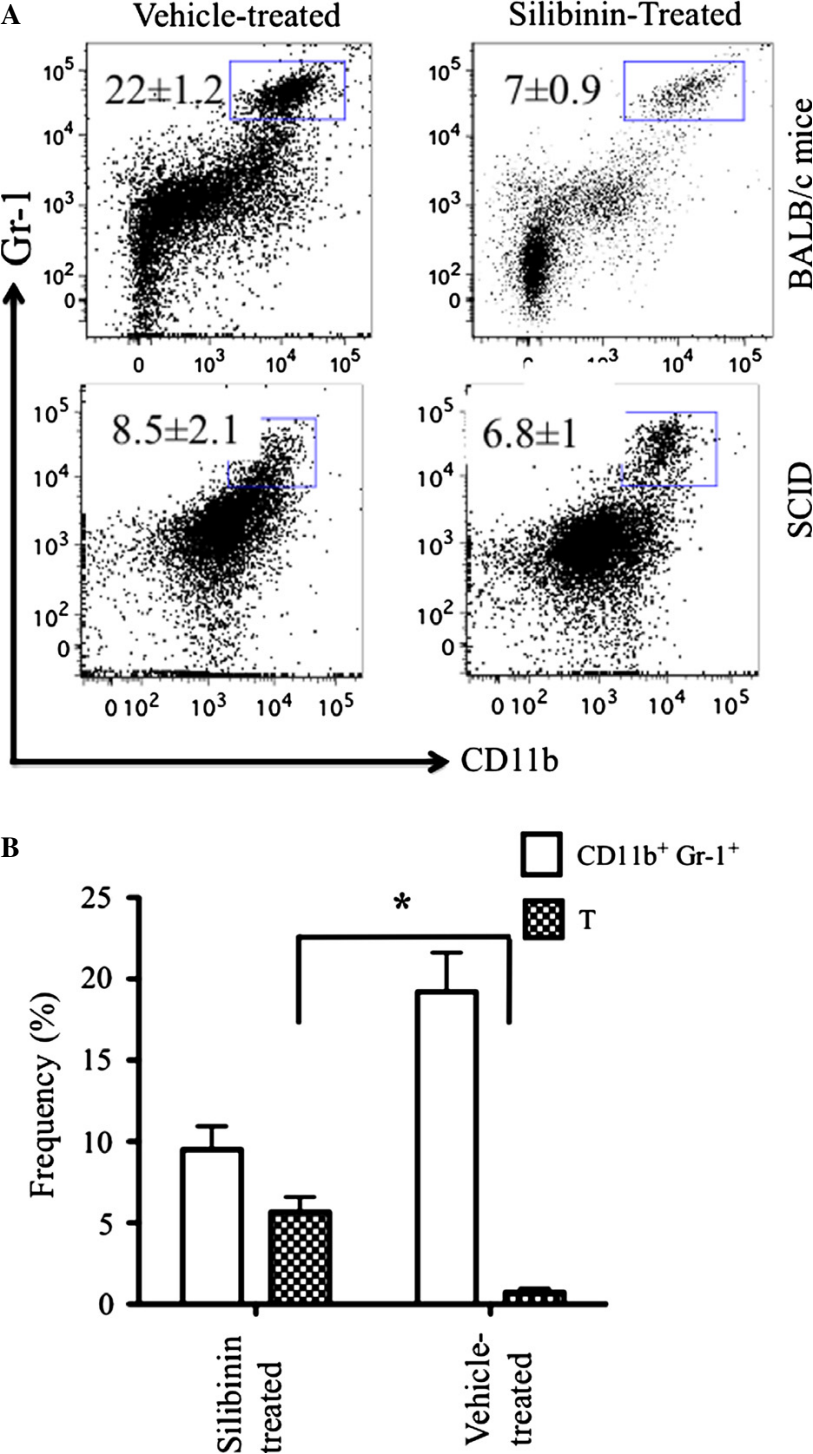
Silibinin treatment decreased MDSCs frequencies and increased T-cell frequencies in the tumor microenvironment of immunocompetent tumor-bearing mice. Immunocompetent BALB/c mice were inoculated with 1 × 10^6^ 4T1-luc tumor cells and began treatment with silibinin or vehicle on day 4. After 10 days, a suspension of tumor cells stained and analyzed for the frequency of 7-AAD negative MDSCs and CD3^+^ T cells by flow cytometry. (A) Representative FACS plots showing the frequency number of MDSCs in the tumor microenvironmnet of immunocompetent BALB/c mice versus immunocompromised SCID mice (average ± SD) on day 10 after tumor inoculation. (B) The percentage of T cells and CD11b^+^ Gr-1^+^ in tumors of vehicle-treated mice versus silibinin-treated mice. The frequency of T cells significantly was higher than vehicle-treated mice (* *P* < 0.05).

Given the in vitro data that physiologically achievable concentrations of silibinin had only a very modest effect on tumor growth in vitro, we next tested whether the observed antitumor effect of silibinin might be an indirect effect of augmenting T-cell mediated antitumor immunity by reducing the number of immunosuppressive MDSCs in the blood and the tumor microenvironment. Previous studies have been shown that MDSCs promote tumor progression in vivo, possibly through a mechanism associated with T-cell dysfunction 33–35. Considering the importance of MDSCs level in regulating the antitumor activity of T cells, we tested whether the antitumor activity of silibinin treatment was dependent upon functional T cells in tumor-bearing animals and the effect of silibinin treatment on MDSCs accumulation in this setting. We next inoculated immunocompromised SCID mice with 4T1-luciferase cells and treated them with either vehicle or silibinin every other day starting on day 4. Untreated BALB/c mice injected with the same number of 4T1 cells were used as an immunecompetent control group. There was no significance difference comparing tumor BLI activity in immunocompromised SCID mice treated with silibinin, compared with vehicle-treated controls (Fig. 4A and B) (*P *>* *0.05). Considering the important role of MDSCs in tumor escape from immune system, we next asked whether silibinin treatment of tumor-bearing SCID mice affected the quantity of MDSCs in blood or tumor following silibinin treatment in immunocompromised SCID mice. While silibinin treatment reduced the content of MDSCs in tumor-bearing BALB/c there was no significant effect on the percentage of MDSCs in the tumor-bearing SCID mice (Figs. 4C and 5A). As the role of MDSCs in regulating T-cell immunity to tumor is well recognized, we next measured the effect of silibinin treatment on the content of T cell in the tumor bed. T-cell infiltration into the tumor increased significantly by day 14 following tumor inoculations in silibinin-treated mice compared with vehicle-treated animals (Fig. 5B).

### Silibinin decreases inflammatory mediators that lead to MDSCs expansion and M2 polarization

**Figure 6:**
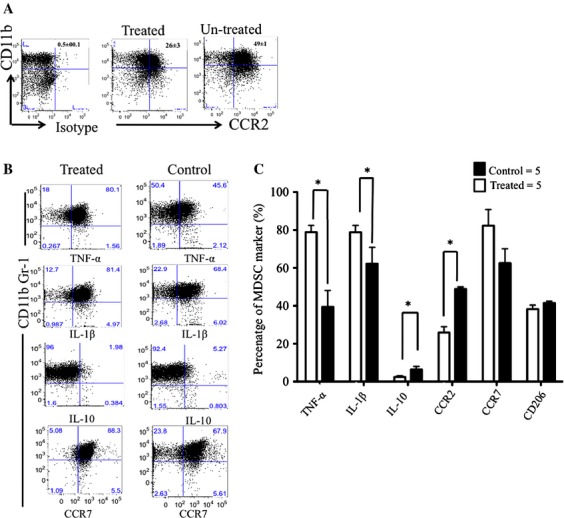
Effect of silibinin on expression of intracellular suppressive mediators on Tumor-derived MDSCs. Normal mice were inoculated s.c. with 1 × 10^6^ of 4T1 tumor cells. Viable CD11b GR-1 double-positive cells harvested from tumor. (A) FACs analysis shows representative frequency of CCR2^+^ MDSCs from two experiments. (B) Representative FACS analysis of CD11b^+^ Gr-1^+^ versus TNF*α*, IL-1*β*, IL-10, and CCR7 in groups treated with silibinin and vehicle. (C) Bars, representative of silibinin-treated (blank bars) and vehicle-treated (solid bars) tumor-derived MDSCs from two experiments. *P < 0.05.

MDSCs are the predominant leukocytes subsets infiltrating solid tumors and CCR2 the most significant chemokine receptor involved in the regulation of the migration of monocytes in both steady state and inflammatory conditions 36. Therefore, we hypothesized that silibinin treatment would decrease CCR2 expression on MDSCs in tumor-bearing animals resulting in lower levels of MDSCs in the blood and in the tumor microenvironment. BALB/c mice were inoculated with 1 × 10^6^ 4T1-luc cells, tumor tissues were harvested and single-cell suspensions analyzed on day 28 post tumor inoculation. The expression levels of CCR2 on MDSCs in BALB/c tumors were determined comparing silibinin-treated with vehicle-treated mice (Fig. 6A). Treatment with silibinin decreased CCR2 expression on MDSCs infiltrating the tumor, but not CCR2 expression on MDSCs in the spleen (data not shown), supporting a role for CCR2 expression in tumor-specific homing of MDSCs. As tumors also polarize tumor-associated macrophages (TAMs) toward a protumor (M2) versus an antitumor (M1) phenotype 37,38 1, we further evaluated the effect of silibinin treatment on M1 versus M2 polarization of tumor-derived monocytic MDSCs. We stained tumor-derived MDSCs 14 days after starting silibinin treatment for expression of markers related to M1 phenotype (tumor necrosis factor *α*, IL1*β*, and CCR7) and M2 phenotype (CD206). To evaluate the effect of silibinin on the expression of immunosuppressive cytokines, intracellular expression of IL-10 19, was measured in MDSCs from the tumor microenvironment in 4T1 tumor-bearing mice. Figure 6C summarizes expression levels of TNF*α*, IL1*β*, CCR7, and CD206 based upon their frequency percentages of each markers and demonstrate deviation toward M1 macrophages. Our data indicate that the effect of silibinin on reducing MDSCs infiltration into tumors is likely indirect, due to alteration of migration patterns through decreased expression of CCR2, and that the MDSCs present in the tumors of silibinin-treated animals are polarized toward a M1 macrophage phenotype.

## Discussion

Research over the last three decades has provided convincing evidence that several medicinal herbs may reduce the toxicity associated with cancer chemotherapy and they may have anti-inflammatory properties that inhibit tumor development 39,40^.^ Among these herbs, silibinin has received attention due to its anticancer efficacy 15,41. Although previous data have demonstrated the anticancer effect of silibinin in vitro on different cell lines 16,42,43, these studies did not investigate the effects of silibinin on the immune system and immunoregulatory MDSCs in tumor-bearing mice. A recently published report identified the role of inducible nitric oxide synthase-producing cells (probably MDSCs) in mice with lung tumors treated with silibinin 44. Considering that inflammation in cancer affects MDSCs accumulation, and that silibinin has been identified as an “anti-inflammatory material” 23,37, we hypothesized that silibinin would block accumulation of MDSCs in 4T1 breast tumor-bearing mice.

Our results showed that silibinin-treated animals have lower tumor volume and prolonged survival comparing vehicle-treated animals. In this study, we used BLI as a sensitive tool for monitoring tumor volume in vivo 45. BLI could detect tumors shortly after inoculation, even when as few as 2500 cancer cells were administrated. Additionally, tumor volume measured with BLI was irrespective of tumor size and shape 28. Silibinin treatment was associated with a reduction in blood and tumor-associated MDSCs in wild-type BALB/c mice but not immunocompromised SCID mice, indicating that the antitumor effect of silibinin and its activity in limiting accumulation of MDSCs is mediated indirectly through T cells. The increased numbers of T cells in the tumor microenvironment were associated with a reduction in the numbers of MDSCs in silibinin-treated immunocompetent mice. The hypothesis that the primary antitumor effect of silibinin is mediated by T cells is supported our observation that the initial effect of silibinin treatment was an increase in tumor-infiltrating T cells followed by a reduction in tumor growth and reduction in the accumulation of MDSCs. This suggests that a secondary effect of the increased numbers of tumor-infiltrating T cells is diminished tumor growth and decreased production of inflammatory mediators that recruit MDSCs into the blood and tumor microenvironment.

Comparing silibinin with other candidate therapies that target MDSCs 9,46,47, there are some important points worth highlighting. First, enteral administration of silibinin was well tolerated, with no adverse effect noted following prolonged administration to nontumor-bearing mice. Second, the effects of silibinin administration included both increased frequencies of tumor-infiltrating T cells and decreased numbers of MDSCs in blood and tumor of immunocompetent mice. Third, silibinin treatment reduced tumor growth when compared to a vehicle-treated control group. Thus, silibinin, in contrast to other agents whose action is limited to MDSCs, has pleiotropic effects on both tumor growth and antitumor immunity. Furthermore, previous experiments have shown that silibinin consumption has no substantial adverse effects in humans and rodents at doses as high as 1% (w/w) or 2 g/kg body weight 48–50. However, still there are some points that need to be clarified. The decreased frequency of MDSCs following silibinin treatment persisted, only as long as silibinin continued to be administered. As CCR2 expression has a major role in regulating both the mobilization of monocytes from BM to the blood and homing to tumor 36,51,52, we measured CCR2 expression on MDSCs in the tumor microenvironment of silibinin-treated mice. Our data indicate that silibinin decreases CCR2 expression in MDSCs, thus providing a mechanism for the decreased accumulation of MDSCs in the tumor that results in a decrease in tumor-associated immunosuppression. However, these findings do not exclude the effect of silibinin on other tumor-derived cytokines such as transforming growth factor *β* and vascular endothelial growth factor that are also involved in MDSCs expansion 53. In addition, our data show that silibinin treatment results in deviation of macrophages immune polarization to a M1 phenotype in the tumor microenvironment. Further experiments are needed to determine if the effect of silibinin on MDSCs accumulation in tumor-bearing animals is sufficient to reverse the suppressive effect of the tumor on T cells and to identify the inflammatory mediators that are involved in MDSCs accumulation.

Collectively, the findings in this article support the importance of the immune system in the anticancer activity of silibinin. The central role of immune system in the antitumor activity of silibinin was confirmed by experiments showing lack of activity in tumor-bearing SCID mice. From the data presented here, we can conclude that the decrease in tumor growth and MDSCs accumulation in the blood of silibinin-treated tumor-bearing animals is not primarily due to a direct antitumor effect on 4T1 cells or suppression of MDSCs development in bone marrow, but rather represent an indirect effect of silibinin on T cells in the tumor microenvironment leading to reduced tumor volume, and prolonged survival in silibinin-treated animals. Silibinin is a promising natural compound available for use to be tested in conjunction with other strategies targeting immune checkpoints in cancer 54,55.
